# Genome-Wide Analysis of Codon Usage Bias in *Epichloë festucae*

**DOI:** 10.3390/ijms17071138

**Published:** 2016-07-15

**Authors:** Xiuzhang Li, Hui Song, Yu Kuang, Shuihong Chen, Pei Tian, Chunjie Li, Zhibiao Nan

**Affiliations:** State Key Laboratory of Grassland Agro-Ecosystems, College of Pastoral Agriculture Science and Technology, Lanzhou University, Lanzhou 730000, China; xiuzhang11@163.com (X.L.); songh12@lzu.edu.cn (H.S.); kuangy13@lzu.edu.cn (Y.K.); chenshh13@lzu.edu.cn (S.C.); tianp@lzu.edu.cn (P.T.); zhibiao@lzu.edu.cn (Z.N.)

**Keywords:** codon usage bias, *Epichloë festucae*, grass endophyte, natural selection, optimal codons

## Abstract

Analysis of codon usage data has both practical and theoretical applications in understanding the basics of molecular biology. Differences in codon usage patterns among genes reflect variations in local base compositional biases and the intensity of natural selection. Recently, there have been several reports related to codon usage in fungi, but little is known about codon usage bias in *Epichloë* endophytes. The present study aimed to assess codon usage patterns and biases in 4870 sequences from *Epichloë festucae*, which may be helpful in revealing the constraint factors such as mutation or selection pressure and improving the bioreactor on the cloning, expression, and characterization of some special genes. The GC content with 56.41% is higher than the AT content (43.59%) in *E. festucae*. The results of neutrality and effective number of codons plot analyses showed that both mutational bias and natural selection play roles in shaping codon usage in this species. We found that gene length is strongly correlated with codon usage and may contribute to the codon usage patterns observed in genes. Nucleotide composition and gene expression levels also shape codon usage bias in *E. festucae*. *E. festucae* exhibits codon usage bias based on the relative synonymous codon usage (RSCU) values of 61 sense codons, with 25 codons showing an RSCU larger than 1. In addition, we identified 27 optimal codons that end in a G or C.

## 1. Introduction

The introduction the genetic code refers to the sequences of DNA and RNA nucleotides that determine amino acid sequences in proteins. The genetic code comprises 64 codons encoding 20 amino acids. Therefore, some amino acids are encoded by more than one codon. Different codons that encode the same amino acid are termed synonymous codons. Although their corresponding tRNAs may differ in speed due to their relative abundances, all codons are recognized by the ribosome. The most amino acids can be encoded by more than one codons. Synonymous codons do not appear randomly throughout the genome, however, a phenomenon that is referred to as codon usage bias [1,2]. Differences in codon usage can modulate the efficiency and accuracy of protein production while maintaining the same protein sequence.

Studies of codon usage have determined that several factors may influence codon usage patterns, including mutational bias and natural selection. Analysis of codon usage patterns sheds light on the molecular biology of gene regulation, gene expression, secondary protein structure, selective transcription, and the external environment. Among these, the major factors that are responsible for codon usage variation among different organisms are compositional constraints under mutational pressure and natural selection [3,4,5,6].

The codon biases of several model species have been analyzed, including *Escherichia coli*, *Drosophila melanogaster*, *Saccharomyces cerevisiae*, *Arabidopsis thaliana*, dengue virus, and humans [1,7,8,9,10,11,12,13,14]. However, little is known about codon usage bias in *Epichloë* endophytes, a group of clavicipitaceous fungi (Clavicipitaceae, Ascomycota) that live in systemic symbioses with cool-season grasses of the subfamily Pooideae [15,16]. This mutualistic symbiotic association confers on the host a number of bioprotective benefits by producing secondary fungal metabolites that alter host metabolism [17]. While *Epichloë* endophytes provide many benefits for their hosts, including both abiotic and biotic factors [18,19], as well as enhanced growth [20,21], the alkaloids produced by the symbiosis can cause health problems for grazing livestock [22]. *E. festucae* is a biotrophic fungus that systemically colonizes the aerial tissues of the cool-season grasses *Festuca*, *Lolium*, and *Koeleria* spp. to form a highly structured symbiotic hyphal network [23,24,25,26]. *E. festucae* has been adopted as the model experimental system for the study of the cellular mechanisms underlying endophyte–grass symbiotic interactions [24,26]. Recently, the genome sequences of two *E. festucae* strains were released [27]. In this study, we analyzed the codon usage bias of *E. festucae* and its relationship with other genome features. Our results lay the groundwork for analyses of genetic evolution in *E. festucae*.

## 2. Results and Discussion

Codon usage bias in genes is an important evolutionary parameter and has been increasingly documented in a wide range of organisms from prokaryotes to eukaryotes. Two theories—neutral evolution and natural selection—have been used to explain the origin of codon usage bias [3,28,29]. If a synonymous mutation occurs at the third codon position, it should result in a random codon choice, with GC and AT being substituted proportionally among the degenerate codons in a gene [30,31]. In contrast, if translational selection pressure influences codon usage, the bias should be significantly positively correlated with expression levels, with some translation-preferred codons appearing more frequently than others. Previous studies have demonstrated that genes within a species often share similar codon usage patterns, though a few species, such as *Bacillus subtilis*, appear to refute this [32].

### 2.1. Base Composition of E. festucae

**Figure 1:**
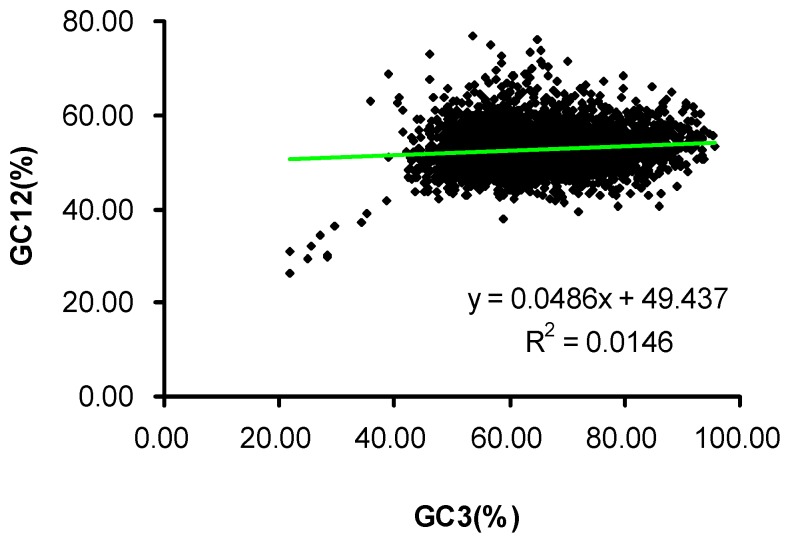
Neutrality plots (GC12 vs. GC3).GC12 stands for the average value of GC content in the first and second position of the codons (GC1 and GC2). While GC3 refers to the GC content in the third position. The solid line is the linear regression of GC12 against GC3, R^2^ = 0.0146, *p* < 0.01.

The GC content for the total 4870 genes varies from 46.43% ± 5.80% (GC2) to 64.11% ±10.16% (GC3), with a mean value of 56.41% ± 4.6% being distributed mainly between 24.80% and 73.00% (Table 1), the GC12 being distributed mainly between 40.00% and 60.00% (Figure 1). The greatest differences of GC content are found in GC2 (46.43% ± 5.80%) and GC3 (64.11% ± 10.16%), where most synoymous mutations occur [33].

### 2.2. Neutrality Plot

A neutrality plot revealed the relationship between GC12 and GC3 (Figure 1), which may reflect the mutation-selection equilibrium that shapes codon usage in *E. festucae*. The neutrality plot shows that *E. festucae* genes exhibit a wide range of GC3 values, ranging from 21.82% to 91.67%. There was a significant positive correlation between GC12 and GC3 (r = 0.121, *p* < 0.01). The slope of the regression line for all coding sequences was 0.0486. The results reveal that the effect of mutation pressure is only 4.86%, it means the codon bias was affected a little by neutral evolution, while the influence from other factors, for example natural selection, is 95.14%. In the genome of *E. festucae*, there was a significant correlation (*p* < 0.01) and the regression coefficient was 0.121. This significantly positive correlation in neutrality plots indicated that the effect on the GC contents by the intra genomic GC mutation bias was similar at all three codon positions [33]. Accordingly, mutation pressure (nucleotide bias) only plays a minor role in shaping the codon bias, whereas natural selection probably dominates the codon bias. These results suggest that an effect of natural selection is present at all codon positions.

### 2.3. Association between Effective Number of Codons (ENC) and GC3s

The ENC in *E. festucae* genes ranged from 26.02 to 61.00, with an average of 51.58. Among the 4870 genes, only 132 genes exhibited high codon bias (ENC < 35), indicating that *E. festucae* genes, in general, reflect random codon usage without strong codon bias.

**Figure 2:**
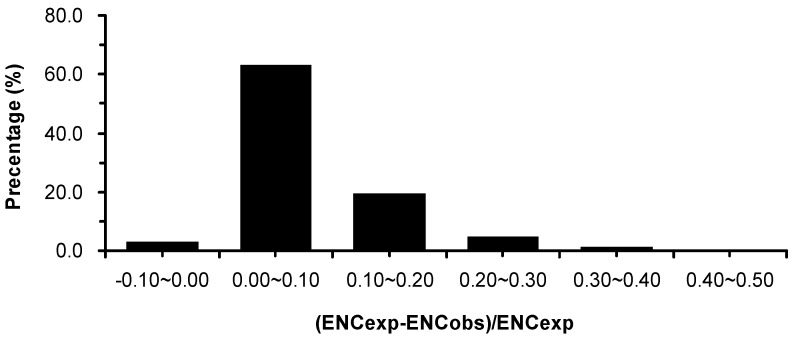
The frequency distribution of effective number of codons (ENC) ratio.

We estimated the difference between the observed and the expected ENC values for all genes using a plot of the frequency distribution of (ENC_exp_ − ENC_obs_)/ENC_exp_ (Figure 2). Most genes appear in the 0.0–0.1 range, suggesting that most observed ENC values are smaller than the ENC values expected based on the GC3s. These results show that *E. festucae* codon usage can be predicted from GC3s and that mutation plays a role in shaping codon usage.

**Figure 3:**
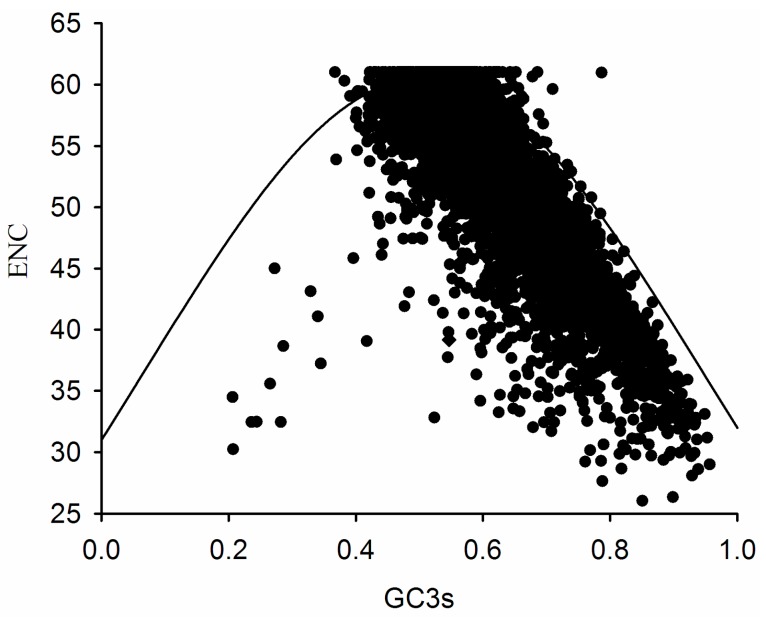
Effective number of codons ENC-plot showing relationship between ENC and GC3s.

An ENC plot was generated to explore the influence of GC3s on codon bias in *E. festucae*. If a gene is located on the expected curve, the codons of that gene are no bias. In this study, most ENC values were lower than expected and were located right below the curve (Figure 3), indicating that other factors, combined with mutation pressure, affects codon usage.

Kawabe and Miyashita [34] reported that the width of the GC3s distribution might be related to variation in the strength of directional selection against mutation pressure. In *E. festucae*, the GC3s distribution was between 0.4 and 1.0, indicating that *E. festucae* mainly evolved by mutation pressure.

The ENC is often used in population genetics research to measure the overall codon bias for an individual gene without knowledge of the optimal codons or a reference set of highly-expressed genes. From the ENC plot, a comparison of the observed distribution of genes with the expected distribution based on GC3s can reveal whether the codon biases of genes are influenced by mutation, but the mutation might not be the unique factor [30].

If a given gene is only subject to GC composition/mutation constraints, it will lie just above or below the standard curve. However, if a particular gene is under selective pressure for high expression, its ENC value will deviate more strongly from the expected value, and it will lie significantly below the curve. In the ENC plot, at a GC3 of approximately 0.4, there were some genes that displayed a more biased codon usage than expected based on the respective GC3s.

The translation efficiency constrains codon choice, which the frequency of codon usage is positively correlated with tRNA availability. The degree of codon usage bias is related to the level of gene expression, with highly-expressed genes exhibiting greater codon bias than lowly-expressed genes. Thus, highly-expressed genes reduce the use of these codons under the selection pressure as far as possible [35,36,37]. While the ENC values of high-expression genes will deviate more strongly from the expected value, this indicates that the translation efficiency is associated with small ENC.

### 2.4. Correlations between Codon Usage Bias, Hydrophobicity, Aromaticity, and Gene Length in E. festucae

To determine the relationship between relative codon bias and nucleotide composition in *E. festucae*, relationships between codon usage bias and hydrophobicity, aromaticity, and gene length were determined using multivariate correlation analysis (Table 2). The results showed that neither the GRAVY (General average hydropathicity) values nor the Aromo values were significantly correlated with GC3s. Aromo and GRAVY values did, however, exhibit significant negative correlations with ENC values (r = −0.034, *p* < 0.05; r = −0.164, *p* < 0.01, respectively), indicating that Aromo and GRAVY values are negatively correlated with codon usage bias in *E. festucae*. Gene length was positively correlated with ENC values (r = 0.227, *p* < 0.01), suggesting that gene length may contribute to codon usage bias. The ENC values were significantly positively correlated with the first axis (r = 0.836, *p* < 0.01) and the second axis (r = 0.193, *p* < 0.01) values, but were significantly negatively correlated with the GC3s (r = −0.808, *p* > 0.01) (Table 2). This suggests that ENC may be the main factor shaping codon bias in *E. festucae*.

The CAI (Codon adaptation index), which reflects the gene expression level, exhibited a significant positive correlation with GC (r = 0.018, *p* < 0.01), GC3 (r = 0.265, *p* < 0.01), GC3s (r = 0.266, *p* < 0.01), T3s (r = 0.045, *p* < 0.01), C3s (r = 0.526, *p* < 0.01), GRAVY (r = 0.080, *p* < 0.01), and Aromo (r = 0.144, *p* < 0.01) values. However, the CAI was significantly negatively correlated with the first and second axis and with other nucleotide composition indices (gene length, GC1, GC2, A3s, G3s, and ENC). These results indicate that both nucleotide composition and gene expression level are major factors shaping codon usage bias in *E. festucae*.

To statistically measure the relationship between CAI and codon usage bias in *E. festucae*, the correlation coefficients for the positions of the genes along the first four major axes were analyzed with their indices of amino acid usage in Table 3.

Though the CAI was negatively correlated with the first axis (r = −0.328, *p* < 0.01), the second axis (r = −0.623, *p* < 0.01), and the third axis (r = −0.159, *p* < 0.01), it was not significantly correlated with the fourth axis (r = 0.005, *p* > 0.05). GRAVY values were positively correlated with Aromo values (r = 0.420, *p* < 0.01) and were negatively correlated with all four axes. Aromo values exhibited a significant correlation with the first and second axis.

These results indicate that the most important factor in the amino-acid usage is hydrophobicity, followed by CAI and aromaticity. This provides strong evidence for the inference that selection for translational efficiency of amino acids exists in *E. festucae*.

In addition, the correspondence analysis was used for some specific aromatic amino acids in codon usage in our research, but the effect of amino acid composition on the codon usage of the whole genome needs further study. Some researchers put forward research that ignores the composition of amino acids in the genome, while some study the codon usage, and some very important properties of correspondence analysis, such as rows weighting, are lost in the process, often diminishing the quantity of information to analyze, occasionally resulting in interpretation errors [38].

Four methods of correspondence analysis (CA) have been developed based on three kinds of input data for synonymous codon usage in 241 bacteria genomes: absolute codon frequency, relative codon frequency, and relative synonymous codon usage (RSCU), as well as within-group CA (WCA). The result shows that WCA is more effective than the other three methods in generating axes that reflect variations in synonymous codon usage, and WCA reveals sources that were previously unnoticed in some genomes, such as synonymous codon usage related to replication strand skew [39]. However, these studies are based on bacteria and some other prokaryote microbiology research, so we are not sure whether the WCA is also the best in eukaryotic organisms, such as fungi. In our study, we just select the CA based on the RSCU, which is widely used to identify major sources of variation in synonymous codon usage. As this is a first study of the codon usage in fungi of *Epichloë* endophytes, we want to find some common codon usage patterns in this fungi. It is necessary to compare more genomes between fungi and bacteria in future studies.

### 2.5. Optimal Codons in E. festucae

We found that *E. festucae* exhibits weak codon biases based on the RSCU values of the 61 sense codons (Table 4). Twenty-five codons were frequently used, such as CUC (RSCU = 1.84) and GGC (RSCU = 1.79), encoding Leu and Gly, respectively. Most frequent codons ended in a G or C, such as UUC, UUG, AUC, and GUC.

The total putative optimal codons of *E. festucae* are presented in Table 5. There is the synonymous codon of each amino acid, and the RSCU values and codon numbers with corresponding “high” and “low” expression date dataset behind each synonymous codon. The number of the synonymous codons of each amino acid is different, such as Ser, were encoded by four codons (UCU, UCC, UCA, and UCG); behind the corresponding codons are the RSCU values and codon numbers with corresponding “high” and “low” expression date dataset, respectively.

There are 27 optimal codons that end in a G (14/27) or C (13/27). This suggests that the preferred codons of *E. festucae* may be related to the GC content at third positions. There are three optimal codons (AGG, CGC, and CGG) encoding the amino acid Arg and two optimal codons each that encode Ala, Thr, Pro, Ser, Val, and Leu. These codons were significantly correlated with translation levels and may be useful in the design of degenerate primers and investigations into the evolutionary history of *E. festucae*.

Similarly to *E. festucae*, the optimal codons of *Aspergillus nidulans* [40], *Oryza sativa* [41], *Triticum aestivum* [33], *Zea mays* [42], and other higher plant nuclear genomes end in G or C, though this differs from results from *E. coli*, *B. subtilis*, *Dictyostelium discoideum*, *D. melanogaster*, *Schizosaccharomyces pombe*, *S. cerevisiae*, and other *Saccharomyces* spp. [7,43]. Close to one-third of all optimal codons end in a uracil, while others end in cytosine or guanine. This phenomenon may be related to their origin and relatives.

In summary, codon usage bias in *E. festucae* was found to be relatively weak and affected by nucleotide composition, mutational pressure, natural selection, and gene expression level. However, natural selection may play a major role in shaping codon usage variation, manifesting itself in weaker codon usage bias. In addition, the codon preferences of *E. festucae* were more biased than those of *A. thaliana*, *E. coli*, or *Caenorhabditis elegans* [44].

Currently, no complete *Epichloë* sp. mitochondrial genome is available in GenBank. As more complete mitochondrial and nuclear genomes of *Epichloë* species are released, further comparative analyses will be possible, allowing for investigation of the genetic and environmental constraints that influence codon usage patterns at the intra- and inter-species levels. In addition, because *Epichloë* is an endophytic fungus, different strains possess different host specificities. Comparing the differences in codon usage of different *Epichloë* strains from the same species may explain these host specificities. Moreover, comparisons of codon usage between the mitochondrial and nuclear genomes in the same *Epichloë* strain may enable exploration into the mechanism of interaction between *Epichloë* endophytes and host grasses.

## 3. Materials and Methods

The complete genome sequences of *E. festucae* (E2368, version 4) were obtained from Genome Projects at University of Kentucky [45]. CDS (Coding DNA sequences) were downloaded from GenBank [46]. To improve the quality of sequences and minimize sampling errors, CDS were filtered based on the following considerations: (i) the presence of a start codon at the beginning and a stop codon at the end of each CDS was required; (ii) each CDS had to be greater than 300 nucleotides in length; and (iii) duplicated sequences (exact matches) were detected and excluded from the dataset. As a result, 4870 CDS were used for further analysis.

### 3.1. Indices of Codon Usage and Synonymous Codon Usage Bias

The GC3s value is defined as the proportion of GC nucleotides at the third (variable) coding position of synonymous codons. It is a useful parameter for evaluating the degree of base composition bias.

Similarly, A3s, G3s, C3s, T3s, and GC3s values can also be deduced by analogy to quantify the usage of each base at synonymous third codon positions. The GC content of each full-length gene, as well as at first, second, and third codon positions (GC, GC1, GC2, and GC3, respectively) were also calculated. GC12 represents the average of GC1 and GC2 and was used for neutrality plot analysis.

Codon adaptation index (CAI) values are often used to measure the extent of bias toward codons that are known to be preferred in highly expressed genes [47]. With values ranging from 0 to 1.0, the higher the value is, the higher the expression level will be.

The effective number of codons (ENC) value, ranging from 20 to 61, is used to measure the magnitude of codon bias in individual genes. This is also a measure of the unevenness of use of codons across all amino acids in a protein. It is worth noting that ENC values are affected by base composition. If all codons for each amino acid were used equally (completely random usage), the ENC would be 61, while if a single codon was used for each amino acid, the ENC would be 20 [30].

The relative synonymous codon usage (RSCU) is the ratio of the observed frequency of codons relative to the expected frequency of the codon under a uniform synonymous codon usage. An RSCU value equal to 1 reflects that codon use is not biased. RSCU values less than 1.0 occur when the observed frequency is less than the expected frequency, and vice versa [48].

General average hydropathicity (GRAVY) values represent the sum of the hydropathy values of all amino acids in the gene product divided by the number of residues in the sequence [49]. The more negative the GRAVY value, the more hydrophilic the protein, while the more positive the GRAVY value, the more hydrophobic the protein.

Aromo values denote the frequency of aromatic amino acids (Phe, Tyr, Trp) in the hypothetical translated gene product. The Aromo and GRAVY values have been used to quantify the major correspondence analysis (COA) trends in the amino acid composition of *E. coli* genes [40].

### 3.2. ENC Plot

The ENC plot (a plot of ENC vs. GC3s) is a strategic investigation into patterns of synonymous codon usage, providing a visual display of the main features of codon usage patterns for a number of genes. Values of ENC were always within the range from 20 (only one codon effectively used for each amino acid) to 61 (codons used randomly). The expected ENC values were calculated as follows [30]:

ENCexp = 2 + S + (29/(S^2^ +(1 − S^2^))) where S is the frequency of G + C (i.e., GC3s).

### 3.3. Neutrality Plot

A neutrality plot [50] can be used to analyze factors influencing codon usage patterns and biases, including estimation and characterization of the relationships between GC12 and GC3.

A neutrality plot regression with a slope of 0 indicates no effects of directional mutation pressure (complete selective constraints), while a slope of 1 is indicative of complete neutrality [50].

### 3.4. Determination of Optimal Codons

The independent optimal codon index can be used as a standard to distinguish between strong and weak translation-coupled biases in datasets. In this study, we ordered the sequences by their ENC ratio values. Using the 5% of sequences from either end of the ordered dataset, we formed two subsets: the “high bias” dataset comprised genes with higher overall ENC ratios, suggesting that their observed ENC values were far from those expected based on GC content, while the “low bias” dataset comprised genes with the lowest ENC ratios [33]. When the difference between the RSCU of “high bias” and “low bias” dataset (ΔRSCU) was larger than 0.08, the corresponding codon was defined as the optimal codon [41].

### 3.5. Correspondence Analysis of RSCU

Correspondence analysis (COA) is a widely used method in multivariate statistical analysis of codon usage patterns. While there are a total of 59 synonymous codons (excluded three termination codons, methionine (Met) and tryptophan (Trp)), in order to generate a COA of RSCU, the degrees of freedom were reduced to 40 after removing variations caused by the unequal usage of amino acids [44].

### 3.6. Software Used

Using Mobyle server [51], including Codon W (Ver.1.4.4) [52], we selected yeast as the model in this research. CHIPS [53] and CUSP [54] were used to calculate the indices of codon usage bias.

### 3.7. Statistical Analysis

Correlations between codon usage and various indices were carried out using SPSS 19.0 (SPSS Inc., Chicago, IL, USA). Effects were corrected for multiple testing with a Tukey-Kramer test, with *p* ≤ 0.01 and *p* ≤ 0.05 as significance levels, respectively [55]. All analyses were performed with SPSS, version 22.0 and GraphPad Prism 5 (GraphPad Software, San Diego, CA, USA).

## Figures and Tables

**Table 1:** Base composition, ENC, Gravy, and Aromo of codons from *Epichloë Festucae.*

Class	Genes	Codons	GC (%)	GC1 (%)	GC2 (%)	GC3 (%)	GC3s (%)	T3s (%)	C3s (%)	A3s (%)	G3s (%)	Gravy	Aro	ENC	CAI
Total	4870	2498255	56.41 ± 4.60	58.68 ± 5.30	46.43 ± 5.80	64.11 ± 10.16	62.86 ± 10.52	23.91 ± 7.41	43.85 ± 9.99	21.13 ± 7.5	33.66 ± 7.37	−0.41 ± 0.37	0.07 ± 0.02	51.58 ± 7.14	0.22 ± 0.04

**Table 2:** Correlation coefficients between the positions of genes along the first two major axes with index of total genes’ codon usage and synonymous codon usage bias.

	Length	GC	GC1	GC2	GC3	GC3S	A3S	T3S	C3S	G3S	GRAVY	AROMO	ENC	CAI	AXIS1
GC	−0.191 **														
GC1	−0.041 **	0.568 **													
GC2	−0.069 **	0.443 **	0.085 **												
GC3	−0.199 **	0.808 **	0.201 **	−0.013											
GC3S	−0.200 **	0.816 **	0.214 **	−0.004	0.998 **										
A3S	0.189 **	−0.710 **	−0.181 **	−0.028 *	−0.854 **	−0.857 **									
T3S	0.160 **	−0.753 **	−0.196 **	−0.092 **	−0.868 **	−0.868 **	0.508 **								
C3S	−0.152 **	0.635 **	0.120 **	−0.063 **	0.835 **	0.838 **	−0.760 **	−0.671 **							
G3S	−0.117 **	0.393 **	0.168 **	−0.194 **	0.557 **	0.555 **	−0.383 **	−0.530 **	0.060 **						
GRAVY	−0.065 **	0.033 *	−0.180 **	−0.138 **	0.217 **	0.207 **	−0.336 **	−0.114 **	0.198 **	−0.063 **					
AROMO	−0.040 **	−0.195 **	−0.412 **	−0.317 **	0.130 **	0.106 **	−0.129 **	−0.028	0.163 **	0.004	0.420 **				
ENC	0.227 **	−0.673 **	−0.211 **	−0.008	−0.799 **	−0.808 **	0.786 **	0.608 **	−0.807 **	−0.252 **	−0.164 **	−0.034 *			
CAI	−0.024	0.108 **	−0.015	−0.193 **	0.265 **	0.266 **	−0.455 **	0.045 **	0.526 **	−0.176 **	0.080 **	0.144 **	−0.402 **		
AXIS1	0.195 **	−0.813 **	−0.231 **	−0.023	−0.970 **	−0.972 **	0.850 **	0.830 **	−0.884 **	−0.436 **	−0.182 **	−0.087 **	0.836 **	−0.328 **	
AXIS2	−0.050 **	0.119 **	0.034 *	0.042 **	0.120 **	0.115 **	0.177 **	−0.374 **	−0.345 **	0.690 **	−0.054 **	−0.025	0.193 **	−0.613 **	0.000

** *p* < 0.01; * *p* < 0.05.

**Table 3:** Correlation coefficients between the positions of genes along the first four major axes with an index of total genes’ amino acid usage.

	CAI	GRAVY	Aromo	Axis1	Axis2	Axis3
Gravy	0.080 **					
Aromo	0.144 **	0.420 **				
Axis1	−0.328 **	−0.182 **	−0.087 **			
Axis2	−0.613 **	−0.054 **	−0.025 *	0.000		
Axis3	−0.159 **	−0.056 **	0.007	0.000	0.001	
Axis4	0.005	−0.008	−0.036 **	−0.003	−0.001	0.002

** *p* < 0.01; * *p* < 0.05.

**Table 4:** Codon usage of *Epichloë festucae.*

Amino Acid	Codon	Total Count	RSCU
**Phe**	UUU	31,361	0.71
	UUC	53,807	1.28
**Leu**	UUA	8539	0.22
	UUG	39,289	1.05
	CUU	32,567	0.86
	CUC	63,009	1.84
	CUA	15,908	0.42
	CUG	57,144	1.61
**Ile**	AUU	36,953	0.97
	AUC	56,383	1.61
	AUA	14,980	0.42
**Met**	AUG	56,564	1.00
**Val**	GUU	33,011	0.83
	GUC	63,583	1.69
	GUA	15,230	0.39
	GUG	41,264	1.08
**Tyr**	UAU	21,165	0.68
	UAC	39,027	1.29
**Cys**	UGU	10,231	0.59
	UGC	20,393	1.25
**His**	CAU	27,393	0.81
	CAC	36,289	1.16
**Gln**	CAA	44,072	0.80
	CAG	59,569	1.19
**Asn**	AAU	32,251	0.70
	AAC	55,829	1.29
**Lys**	AAA	37,695	0.63
	AAG	80,172	1.37
**Asp**	GAU	60,666	0.79
	GAC	87,756	1.21
**Glu**	GAA	60,978	0.76
	GAG	89,320	1.23
**Ser**	UCU	33,326	0.87
	UCC	44,291	1.33
	UCA	30,242	0.76
	UCG	39,274	1.14
**Pro**	CCU	35,400	0.86
	CCC	49,710	1.37
	CCA	35,372	0.83
	CCG	36,167	0.93
**Thr**	ACU	27,362	0.71
	ACC	46,502	1.30
	ACA	31,612	0.81
	ACG	40,791	1.17
**Ala**	GCU	49,281	0.84
	GCC	88,269	1.58
	GCA	44,142	0.75
	GCG	45,406	0.83
**TER**	UGA	2445	1.39
	UAA	1271	0.74
	UAG	1529	0.87
**Trp**	UGG	34,101	0.94
**Arg**	CGU	20,731	0.73
	CGC	40,880	1.55
	CGA	33,836	1.13
	CGG	23,806	0.86
	AGA	24,225	0.84
	AGG	23,489	0.89
**Gly**	GGU	35,358	0.75
	GGC	79,015	1.79
	GGA	35,677	0.78
	GGG	28,001	0.67
**Ser**	AGU	21,367	0.57
	AGC	46,172	1.32

**Codon** indicates synonymous codons; **Total Count** indicates the number of the synonymous codons; **RSCU** indicates relative synonymous coden usage, the preferentially-used codons are underlined.

**Table 5:** Optimal codons of genes in *E. festucae.*

Amino Acid	Codon	High RSCU	N	Low RSCU	N
**Phe**	UUU	0.41	1246	0.97	4176
	UUC *	1.59	4874	1.03	4414
**Leu**	UUA	0.03	86	0.54	2081
	UUG	0.55	1375	1.27	4893
	CUU	0.29	744	1.22	4713
	CUC *	2.90	7320	1.10	4257
	CUA	0.14	358	0.71	2725
	CUG *	2.08	5252	1.16	4456
**Ile**	AUU	0.57	1339	1.20	4799
	AUC *	2.24	5277	1.13	4511
	AUA	0.19	442	0.68	2704
**Met**	AUG	1.00	3940	1.00	5668
**Val**	GUU	0.35	1025	1.18	4457
	GUC *	2.39	7011	1.21	4561
	GUA	0.11	322	0.63	2379
	GUG *	1.16	3396	0.97	3649
**Tyr**	UAU	0.29	651	1.03	3182
	UAC *	1.71	3910	0.97	2979
**Ser**	AGU	0.24	488	0.76	3234
	AGC *	1.61	3266	1.05	4495
**His**	CAU	0.42	875	1.11	3946
	CAC *	1.58	3322	0.89	3180
**Gln**	CAA	0.42	1184	1.07	6735
	CAG *	1.58	4457	0.93	5867
**Asn**	AAU	0.30	808	0.98	4824
	AAC *	1.70	4557	1.02	4978
**Lys**	AAA	0.29	1091	0.94	5763
	AAG *	1.71	6306	1.06	6539
**Asp**	GAU	0.40	1941	1.03	8086
	GAC *	1.60	7765	0.97	7678
**Glu**	GAA	0.39	1802	1.03	8278
	GAG *	1.61	7396	0.97	7846
**Ser**	UCU	0.40	818	1.22	5215
	UCC *	1.96	3989	0.89	3811
	UCA	0.31	635	1.17	5018
	UCG *	1.48	2998	0.91	3878
**Pro**	CCU	0.43	1030	1.10	4937
	CCC *	2.13	5100	0.81	3633
	CCA	0.32	763	1.27	5704
	CCG *	1.12	2689	0.81	3651
**Thr**	ACU	0.28	685	1.04	4130
	ACC *	1.80	4428	0.91	3615
	ACA	0.38	941	1.20	4774
	ACG *	1.54	3796	0.86	3422
**Ala**	GCU	0.38	1665	1.14	6365
	GCC *	2.29	9960	1.05	5840
	GCA	0.34	1462	1.10	6141
	GCG *	0.99	4333	0.70	3903
**Cys**	UGU	0.29	322	0.95	1626
	UGC *	1.71	1869	1.05	1790
**Trp**	UGG	1.00	2561	1.00	3365
**Gly**	GGU	0.39	1322	0.96	4175
	GGC *	2.55	8641	1.35	5864
	GGA	0.37	1263	1.03	4480
	GGG	0.69	2353	0.65	2813
**Arg**	AGA	0.45	825	1.17	3566
	AGG *	1.29	2390	0.79	2427
	CGU	0.37	680	0.82	2510
	CGC *	2.34	4329	1.02	3113
	CGA	0.50	932	1.41	4306
	CGG *	1.05	1934	0.80	2440
**TER**	UAA	0.62	101	0.79	169
	UAG	0.92	150	0.74	158
	UGA	1.47	240	1.48	317

**Codon** indicates synonymous codons; **N** indicates codon frequency; **RSCU** indicates relative synonymous codon usage; **High** and **Low** indicate the codon usage of 244 genes (5% of the total number of genes) from the top and bottom of the dataset ordered by ENC ratio value, respectively. The optimal codons are indicated with a (*).
